# Peripherally inserted central catheters have a protective role and the effect of fluctuation curve feature in the risk of bloodstream infection compared with central venous catheters: a propensity-adjusted analysis

**DOI:** 10.1186/s12879-022-07265-x

**Published:** 2022-03-26

**Authors:** Yu Lv, Xiaobo Huang, Yunping Lan, Qi Xia, Fuli Chen, Jiayu Wu, Wei Li, Hongrong Cao, Caixia Xie, Luting Li, Hukui Han, Hui Wang, Qian Xiang

**Affiliations:** 1 Healthcare-Associated Infection Management Office, Sichuan Academy of Medical Sciences, Sichuan People’s Hospital, School of Medicine, University of Electronic Science and Technology of China, Chengdu, 610072 Sichuan People’s Republic of China; 2 Intensive Care Unit, Sichuan Academy of Medical Sciences, Sichuan People’s Hospital, School of Medicine, University of Electronic Science and Technology of China, Chengdu, 610072 Sichuan People’s Republic of China; 3 PICC Nursing Center, Sichuan Academy of Medical Sciences, Sichuan People’s Hospital, School of Medicine, University of Electronic Science and Technology of China, Chengdu, 610072 Sichuan People’s Republic of China; 4 Interventional Treatment Center, Sichuan Academy of Medical Sciences, Sichuan People’s Hospital, School of Medicine, University of Electronic Science and Technology of China, Chengdu, 610072 Sichuan People’s Republic of China; 5 Department of Nursing, Sichuan Academy of Medical Sciences, Sichuan People’s Hospital, School of Medicine, University of Electronic Science and Technology of China, Chengdu, 610072 Sichuan People’s Republic of China; 6 Development Department, Chengdu Yiou Technology Co. LTD, Chengdu, 610000 Sichuan People’s Republic of China; 7 Cardiology, Sichuan Academy of Medical Sciences, Sichuan People’s Hospital, School of Medicine, University of Electronic Science and Technology of China, Chengdu, 610072 Sichuan People’s Republic of China

**Keywords:** Peripherally inserted central catheter, Central venous catheter, Bloodstream infection, Propensity score matching, Restricted cubic spline regression

## Abstract

**Background:**

The prevention of peripherally inserted central catheters (PICC)-associated BSI and central venous catheters (CVC)-associated BSI have been a topic of national importance in China. Therefore, we aimed to explore the epidemiological characteristics of central line-associated bloodstream infection (CLABSI), and to evaluate whether PICCs were associated with a protective effect for CLABSI.

**Methods:**

A retrospective cohort study was conducted in teaching hospital in Western China. All adult patients received a CVC or PICC during their hospital stay were included from January 2017 to December 2020. Primary endpoint was CLABSI up to 30 days after CVC or PICC placement. Propensity scores with a 2:1 match was used to account for potential confounders, and restricted cubic spline was used to visualize the risk of CLABSI at different time points during the catheterization.

**Results:**

A total of 224687 devices (180522 PICCs and 45965 CVCs) in 24879 patients were included. The overall incidence was 1.8 CLABSIs per 1000 catheter-days. The odds ratio (OR) value increased day by day after PICC insertion, reached a relatively high point on the 4th day, and decreased from days 5 through 8. From the 9th day of intubation the OR value began to gradually increase day by day again. After covariate adjustment using propensity scores, CVCs were associated with higher risk of CLABSI (adjHR = 3.27, 95% CI 2.38–4.49) compared with PICCs.

**Conclusions:**

PICCs have a protective role and the effect of fluctuation curve feature in CLABSI when compared to CVCs, and the first 8 calendar days after CVC insertion are the acute stage of CVC-associated BSI.

**Supplementary Information:**

The online version contains supplementary material available at 10.1186/s12879-022-07265-x.

## Introduction

Central line-associated bloodstream infections (CLABSIs), one of the common healthcare-associated infections (HAIs), are associated with significant morbidity, mortality, prolonged hospital length of stay (LOS) and excess health care costs [1–3]. Although it is now recognized that CLABSIs are preventable when the evidence-based guidelines are followed [4–6], the surveillance data of the CHN National Clinical Improvement System (NCIS) in recent years show that the incidence of CLABSIs has not decreased significantly [7]. Then in the "national medical quality and safety improvement goals in 2021” issued by the National Health Commission of the People`s Republic of China (PRC) on February 9, 2021 [8], the ninth of the top ten improvement goals is to reduce the incidence of intravascular catheter-related bloodstream infection, and requires that the focus of improvement should be on CLABSI due to central venous catheters (CVCs) and peripherally inserted central catheters (PICCs). Since CLABSI prevention has been a topic of national importance, it is necessary to make in-depth analysis on the characteristics of CVC-associated BSI and PICC-associated BSI. However, there may be different incidence rate, risk factors or other characteristics between the two most commonly used catheters in CLABSI.

Due to the influence of some selective bias or mixed bias, conflicting conclusions commonly found in previous studies about whether there is a difference between CVC-associated BSI and PICC-associated BSI are not conducive to guiding clinical practice [9–11]. Although the risk of CLABSI in different catheters may vary [11, 12], it is recognized that prolonged catheterization can significantly increase the risk of CLABSI no matter what kind of central line is used [13]. At present, linear or curvilinear associations of CLABSI risk over time have not been confirmed, and previous studies have been limited to univariate analysis [13]. Therefore, we conducted this study to assess the association of CLABSI risk over time by restricted cubic spline (RCS), and to evaluate whether PICCs were associated with a protective effect for CLABSI when compared to CVCs after the bias was eliminated by propensity score matching (PSM).

## Material and methods

### Setting

This study was conducted at Sichuan Academy of Medical Sciences, Sichuan People's Hospital, School of Medicine, University of Electronic Science and Technology of China, a 4400-bed tertiary care teaching hospital in Chengdu in the region of Sichuan (Western China). The hospital has an average of 130,000 admissions per year, and comprises 9 (total 167 beds) intensive care units (ICUs), 25 surgical units, 37 medicine wards, 2 transplant units, 1 dialysis centers, 2 traditional Chinese medicine wards and numerous rehabilitation units.

### Study design and patients

We performed a retrospective cohort study from January 2017 to December 2020, to compare the different effects of CLABSI risk between CVCs and PICCs, and to assess the dynamic changes of CLABSI risk during intubation. All adult patients received a CVC or PICC during their hospital stay were included. Patients under the age of 18 years at the time of catheter insertion or those whose catheters were removed within 2 calendar days after placement were excluded. This study was approved by the Ethics Committee of Sichuan Academy of Medical Sciences, Sichuan People's Hospital, School of Medicine, University of Electronic Science and Technology of China.

### Definitions and data collection

Primary endpoint was CLABSI up to 30 days after CVC or PICC placement. CLABSI was defined according with Centers for Disease Control and Prevention (CDC)/National Healthcare Safety Network (NHSN) surveillance definitions and criteria [14]. To accomplish the study objectives, the independent CLABSI monitoring information system, which was developed by the healthcare-associated infections quality control center in Sichuan province, was used to continuously monitor every patient with CVC or PICC. For each patient with CVC and PICC, suspicious CLABSI cases were automatically screened out by the intelligent identification program of the CLABSI monitoring information system if any of the following conditions were met: ① Fever (> 38 °C), ② Hypotension (systolic blood pressure < 90 mmHg and/or diastolic blood pressure < 60 mmHg), ③ Oliguria(< 400 ml/day), ④ Positive blood specimen, ⑤ CLABSI cases that have been prospectively entered into the HAI electronic system by clinicians before retrospective analysis. For this study, four infectious disease specialists revised the electronic medical records of all screened CLABSI cases to check if all NHSN criteria were fulfilled.

The baseline characteristics of patients were retrieved from electronic medical records, including age, gender, principal diagnosis [International Classification of Diseases (ICD)-10 coded] [15], diabetes mellitus, hypertension (systolic blood pressure ≥ 140 mmHg and/or diastolic blood pressure ≥ 90 mmHg) [16], chronic obstructive pulmonary disease (COPD), hemodialysis, mechanical ventilation, urinary catheterization, tracheotomy, surgery, malignancy and community infections. Additional file 1: Table S1 in the Supplement presents the additional data information for our study.

### Prevention of central line-associated bloodstream infections

On the basis of referring to several guidelines in China and the United States [6, 17], we have formulated a bundled strategy for central line-associated bloodstream infections prevention and control (IPC). Additional file 1: Table S2 in the Supplement presents the IPC measures for CLABSI. In order to ensure the implementation of IPC measures, we have established a multi-disciplinary teamwork (MDT) including medical department, nursing department and HAI management department. The division of responsibilities in MDT was as follows: the medical department was responsible for the authorization management of catheterization operators, the nursing department was responsible for the preparation of catheterization related materials, central-line maintenance and quality verification, and the HAI management department was responsible for the coordination, quality control, monitoring, analysis and summary in the whole process.

### Propensity score matching

To minimize the impact of potential bias, a propensity score matching (PSM) was performed in the whole study cohorts using R Package Matching version 4.9–2 (CRAN.R-project.org/pack-age = Matching). The first step, standardized mean differences (SMD) were determined to compare baseline characteristics of patients. And the SMD of less than 0.1 was considered as an indicator of good balance between CVC and PICC group [18]. The second step, the propensity scores were calculated using the logistic regression model that accounted for the baseline characteristics with statistical significance. The third step, using the caliper of width equal to 0.2 of the standard deviation of the legit of the propensity score a k-nearest neighbor algorithm was used to make a 2:1 match without replacement between CVC and PICC group [19]. The fourth step, SMD was used again to test the balance between groups after PSM.

### Statistical analysis

Statistical analysis of the data was performed using STATA version 20.0 (StataCorp. College Station, Texas, USA) and R software (v3.6.1) under RStudio (v1.2.5001). Categorical variables are shown as percentages. The normally distributed variables were presented as mean ± standard deviation and the non-normally distributed variables were expressed as median and interquartile range (IQR). Restricted cubic spline was used to visualize the risk of CLABSI at different time points during the catheterization. Time-to-event analyses were performed using the Kaplan–Meier survival functions to estimate the cumulative CLABSI hazard and the likelihood ratio test was used to compare hazard fraction in different groups. All tests were 2-sided with an α level of 0.05.

## Results

**Fig. 1:**
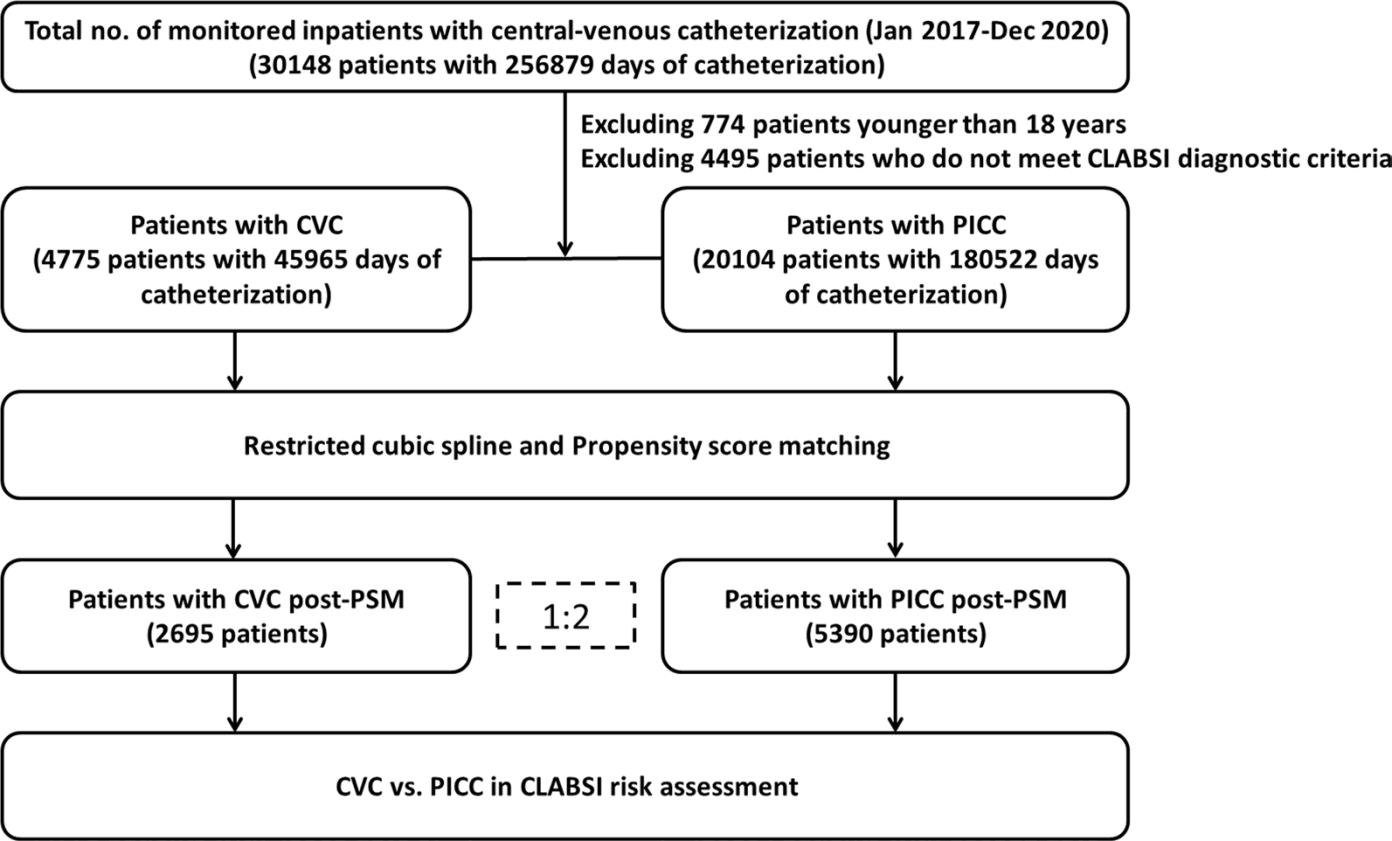
Cases selection algorithm for our study population. We excluded 774 patients younger than 18 years and 4495 patients who did not meet CLABSI diagnostic criteria, resulting in 4775 patients with CVC and 20,104 patients with PICC were included in the analysis

During the study period, a total of 30,148 patients were catheterized for 256,879 days. We excluded 774 patients younger than 18 years and 4495 patients who did not meet CLABSI diagnostic criteria, resulting in 4775 patients with CVC and 20,104 patients with PICC were included in the analysis (Fig. 1). In the whole cohort, 54.3% participants were male, and the mean (SD) ages were 60.69 (17.30) and 56.84 (14.78) years old for those with CVC and PICC, respectively (Table 1). We observed an overall incidence of 1.80 CLABSIs per 1000 catheter-days (407 CLABSIs in 226,487 catheter-days). Compared with patients with PICC, those with CVC had a significantly higher crude CLABSI rate (5.55 events per 1000 catheter-days vs 0.84 events per 1000 catheter-days, *P* < 0.001). The standardized mean differences (SMD) between the groups were over 10% for most investigated baseline characteristics except for year and hemodialysis (Table 1).

**Table 1:** Baseline characteristics

Variable	Pre-PSM, No. (%)			Post-PSM, No. (%)		
	PICC (n = 20,104)	CVC (n = 4775)	SMD	PICC (n = 5390)	CVC (n = 2695)	SMD
Age, mean (SD), y	56.84 (14.78)	60.69 (17.30)	0.239	57.89 (16.27)	58.57 (17.20)	0.041
Male	10,466 (52.1)	3038 (63.6)	0.236	3050 (56.6)	1581 (58.7)	0.042
Year						
2017	3976 (19.8)	1018 (21.3)	0.038	1243 (23.1)	623 (23.1)	0.001
2018	4788 (23.8)	1176 (24.6)	0.019	1462 (27.1)	707 (26.2)	0.020
2019	5373 (26.7)	1218 (25.5)	0.028	1345 (25.0)	665 (24.7)	0.006
2020	5967 (29.7)	1363 (28.5)	0.025	1340 (24.9)	700 (26.0)	0.026
Community Infections	902 (4.5)	1123 (23.5)	0.570	757 (14.0)	414 (15.4)	0.037
Blood Transfusion	6632 (33.0)	3064 (64.2)	0.657	3020 (56.0)	1548 (57.4)	0.028
Urinary Catheterization	8842 (44.0)	3472 (72.7)	0.609	3393 (62.9)	1806 (67.0)	0.085
Hemodialysis	174 (0.9)	65 (1.4)	0.047	56 (1.0)	27 (1.0)	0.004
Mechanical Ventilation	3625 (18.0)	3222 (67.5)	1.154	2453 (45.5)	1325 (49.2)	0.073
Tracheotomy	191 (1.0)	570 (11.9)	0.459	163 (3.0)	115 (4.3)	0.066
Hypertension	5005 (24.9)	1629 (34.1)	0.203	1599 (29.7)	844 (31.3)	0.036
Diabetes mellitus	2418 (12.0)	958 (20.1)	0.220	868 (16.1)	478 (17.7)	0.044
COPD	379 (1.9)	505 (10.6)	0.366	211 (3.9)	129 (4.8)	0.043
Malignancy	9434 (46.9)	471 (9.9)	0.902	572 (10.6)	343 (12.7)	0.066
Liver Failure	152 (0.8)	274 (5.7)	0.284	130 (2.4)	81 (3.0)	0.037
Renal Failure	609 (3.0)	692 (14.5)	0.414	355 (6.6)	250 (9.3)	0.095
Heart Failure	477 (2.4)	418 (8.8)	0.281	259 (4.8)	169 (6.3)	0.064
Respiratory Failure	392 (1.9)	1573 (32.9)	0.895	362 (6.7)	265 (9.8)	0.113
Principal diagnosis						
Certain infectious diseases and parasites	601 (3.0)	258 (5.4)	0.121	291 (5.4)	147 (5.5)	0.002
Tumor	9595 (47.7)	313 (6.6)	1.045	485 (9.0)	289 (10.7)	0.058
Blood and hematopoietic diseases and certain diseases involving immune mechanisms	197 (1.0)	15 (0.3)	0.083	37 (0.7)	15 (0.6)	0.017
Endocrine, nutritional, and metabolic diseases	136 (0.7)	86 (1.8)	0.102	123 (2.3)	55 (2.0)	0.017
Mental and behavioral disorders	9 (0.0)	16 (0.3)	0.067	9 (0.2)	8 (0.3)	0.027
Nervous system diseases	83 (0.4)	113 (2.4)	0.167	74 (1.4)	44 (1.6)	0.021
Eye and appendage diseases	2 (0.0)	0 (0.0)	0.014	0 (0.0)	0 (0.0)	/
Ear and mastoid diseases	2 (0.0)	0 (0.0)	0.014	0 (0.0)	0 (0.0)	/
Circulatory diseases	1892 (9.4)	1098 (23.0)	0.375	1627 (30.2)	738 (27.4)	0.062
Respiratory diseases	730 (3.6)	1054 (22.1)	0.573	484 (9.0)	251 (9.3)	0.012
Digestive diseases	1464 (7.3)	755 (15.8)	0.269	1079 (20.0)	526 (19.5)	0.013
Skin and subcutaneous tissue diseases	11 (0.1)	10 (0.2)	0.043	10 (0.2)	7 (0.3)	0.016
Musculoskeletal system and connective tissue diseases	288 (1.4)	42 (0.9)	0.052	68 (1.3)	33 (1.2)	0.003
Genitourinary diseases	1524 (7.6)	142 (3.0)	0.207	279 (5.2)	128 (4.7)	0.020
Pregnancy, childbirth, and puerperium	99 (0.5)	20 (0.4)	0.011	43 (0.8)	16 (0.6)	0.025
Congenital malformations, deformation, and chromosomal abnormalities	407 (2.0)	11 (0.2)	0.171	18 (0.3)	11 (0.4)	0.012
Abnormal symptoms, signs, clinical and laboratory results, and cannot be classified in other categories	41 (0.2)	28 (0.6)	0.061	40 (0.7)	18 (0.7)	0.009
Injury, poisoning and other external pathogenic factors	723 (3.6)	810 (17.0)	0.451	717 (13.3)	405 (15.0)	0.049
External causes of illness and death	2300 (11.4)	4 (0.1)	0.503	6 (0.1)	4 (0.1)	0.010

A higher incidence of CLABSIs was observed in catheters of femoral (FEM) site as compared to others before (χ^2^ = 386.381, *P* < 0.001) and after PSM (χ^2^ = 39.603, *P* < 0.001). More specifically, the incidence of CLABSIs was as follows: FEM: 6.2% before PSM and 3.8% after PSM, Subclavian (SC): 5.2% before PSM and 3.5% after PSM, Internal Jugular (IJ): 4.5% before PSM and 3.3% after PSM, PICC: 0.8% before PSM and 1.3% after PSM (Table 2).

**Table 2:** CLABSI among the 4 sites of catheterization

Catheter location	Pre-PSM, No. (%)		*P-value*	Post-PSM, No. (%)		*P-value*
	CLABSI (n = 407)	Non-CLABSI (n = 24,472)		CLABSI (n = 164)	Non-CLABSI (n = 7921)	
Catheter insertion sites			< 0.001			< 0.001
PICC	152 (0.8)	19,952 (99.2)		71 (1.3)	5319 (98.7)	
Femoral	74 (6.2)	1128 (93.8)		28 (3.8)	707 (96.2)	
Subclavian	71 (5.2)	1284 (94.8)		25 (3.5)	697 (96.5)	
Internal Jugular	53 (4.5)	1115 (95.5)		22 (3.3)	640 (96.7)	
Unclear	7 (2.7)	255 (97.3)		5 (3.1)	156 (96.9)	
Femoral + Subclavian	46 (6.4)	670 (93.6)		12 (3.2)	366 (96.8)	
Femoral + Internal Jugular	3 (6.0)	47 (94.0)		1 (3.8)	25 (96.2)	
Subclavian + Internal Jugular	1 (4.8)	20 (95.2)		0 (0.0)	10 (100.0)	
Femoral + Subclavian + Internal Jugular	0 (0.0)	1 (100.0)		0 (0.0)	1 (100.0)	

**Fig. 2:**
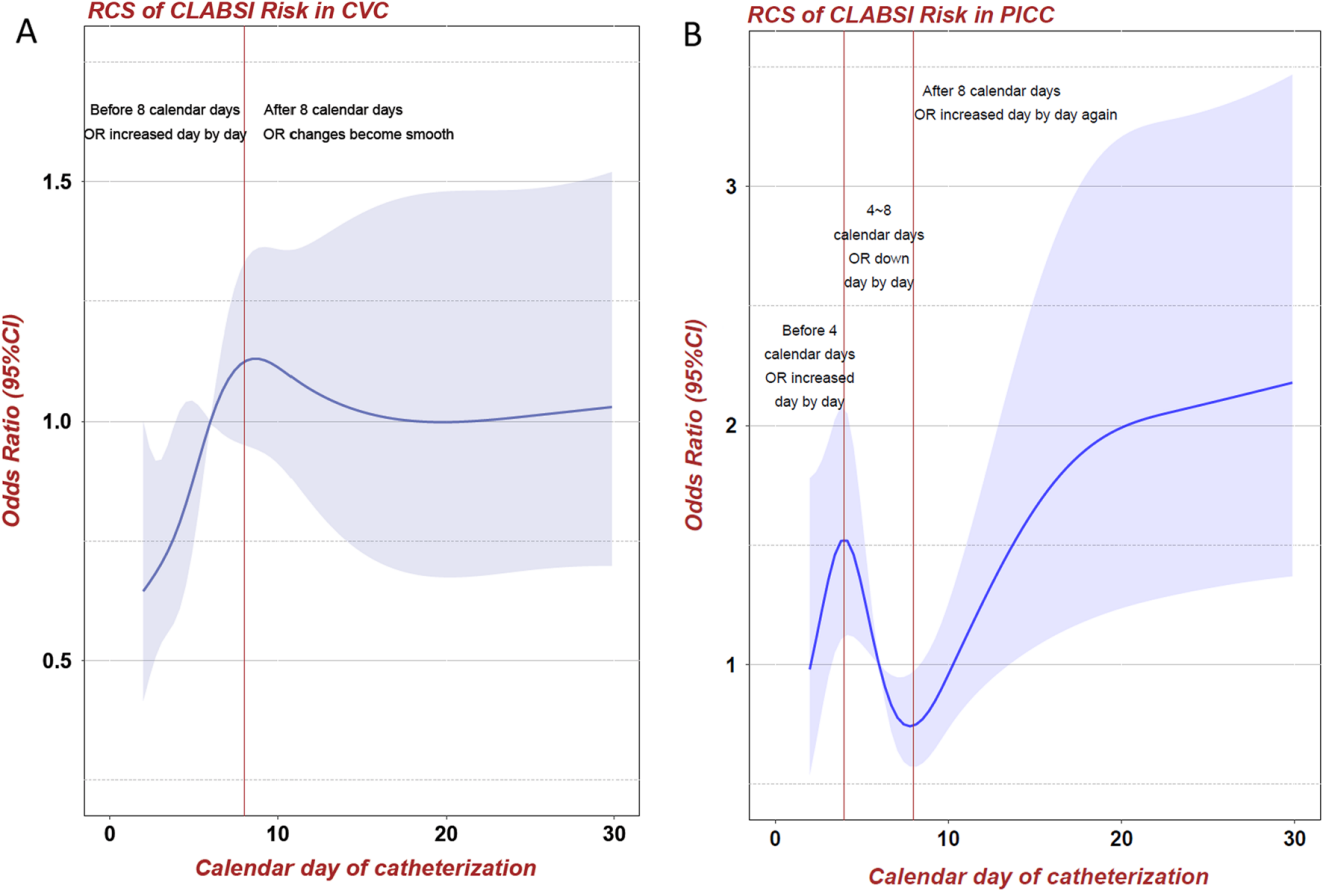
Restricted cubic spline of CLABSI risk. **A** OR value increased rapidly in the first 8 calendar days after CVC insertion. However, after the 8th calendar day the OR value became stable. **B** In the first 4 calendar days after PICC insertion, the OR value increased day by day, reached a relatively high point on the 4th calendar day, and decreased from days 5 through 8. However, after the 8th calendar day the OR value began to increase day by day again

In the first 8 calendar days after CVC insertion, the risk of CLABSI increased rapidly, and the OR value increased day by day. However, after the 8th calendar day the OR value became stable (Fig. 2A). Compared with the CLABSI risk of CVC, the change of CLABSI risk of PICC showed more complex curve characteristics. In the first 4 calendar days after PICC insertion, the OR value increased day by day, reached a relatively high point on the 4th calendar day, and decreased from days 5 through 8. However, after the 8th calendar day the OR value began to increase day by day again (Fig. 2B).

**Fig. 3:**
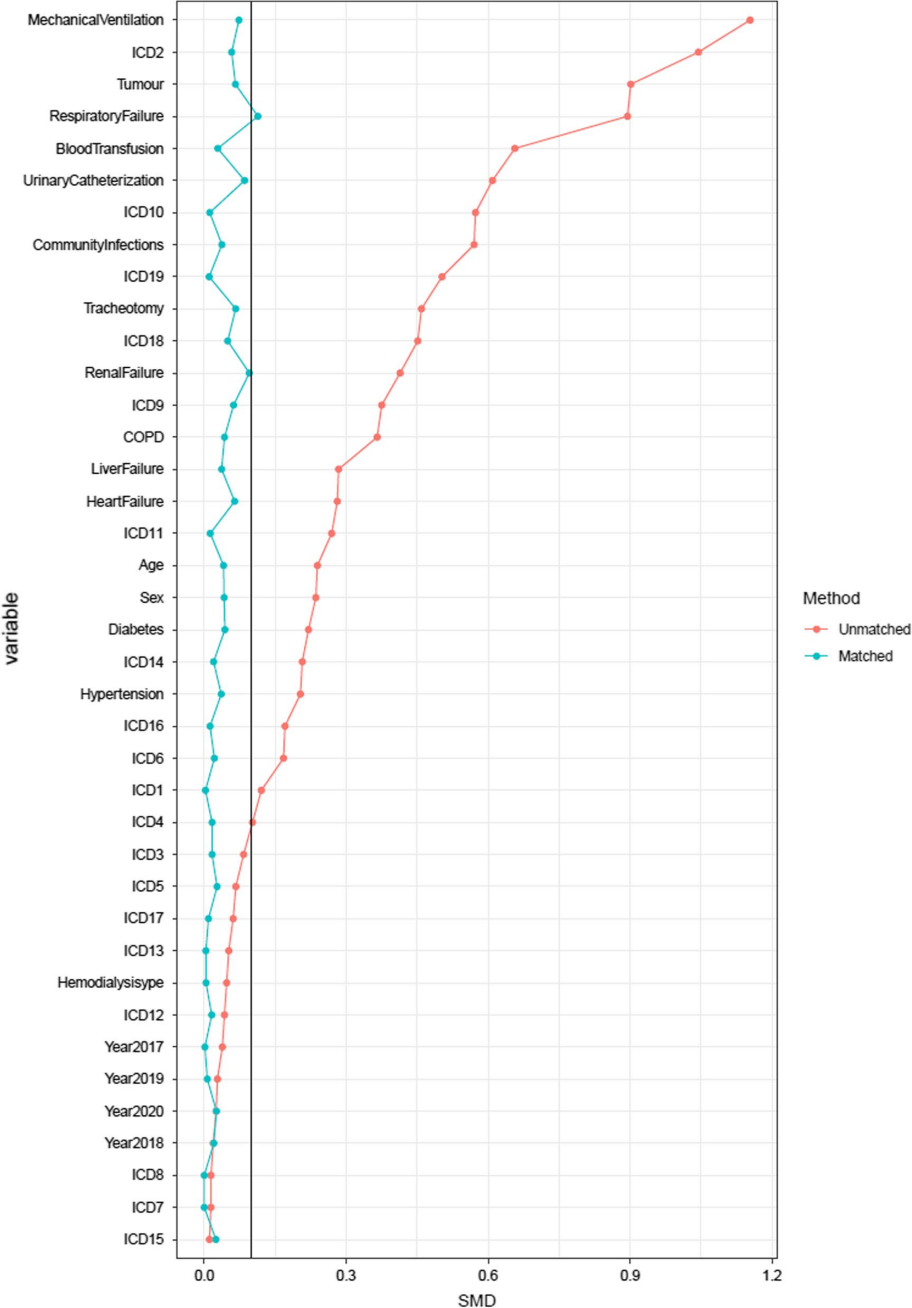
Standardized mean differences before and after matching. PSM improved the balance on the investigated baseline characteristics with all SMD between groups decreasing to 0.1 or less except for the respiratory failure

**Fig. 4:**
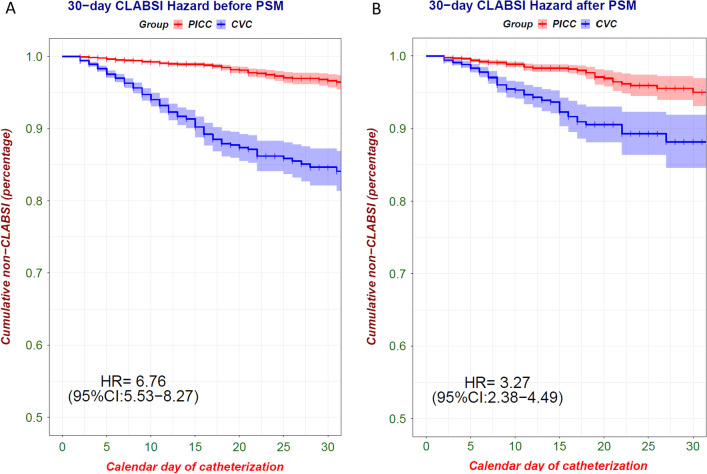
30-day CLABSI hazard before and after propensity score matching. **A** In the whole cohort, patients with CVC had a significantly higher 30-day CLABSI hazard compared with patients with PICC (HR = 6.76, 95% CI 5.53–8.27). **B** In the PSM cohort, patients with CVC also had a significantly higher 30-day CLABSI hazard compared with patients with PICC (adjHR = 3.27, 95% CI 2.38–4.49)

Propensity score matching (PSM) improved the balance on the investigated baseline characteristics with all SMD between groups decreasing to 0.1 or less except for the respiratory failure, which was 0.113 (Table 1 and Fig. 3). After PSM, the final analyzed cohort consisted of 2695 patients with CVC and 5390 patients with PICC (Table 1 and Fig. 1). In the whole cohort, patients with CVC had a significantly higher 30-day CLABSI hazard compared with patients with PICC (HR = 6.76, 95% CI 5.53–8.27, Fig. 4A). In the PSM cohort, patients with CVC also had a significantly higher 30-day CLABSI hazard compared with patients with PICC (adjHR = 3.27, 95% CI 2.38–4.49, Fig. 4B).

## Discussion

This is the first propensity-adjusted study to compare the incidence of CLABSI in PICCs and CVCs in Southwest China. We observed an overall incidence of 1.80 CLABSIs per 1000 catheter-days, higher than the average incidence of 1.00 CLABSIs per 1000 catheter-days of the three-level medical institutions reported in the National Report on the Services, Quality and Safety in Medical Care System [7]. More efforts and actions are needed to further reduce the incidence rate of CLABSI. The prevention of CLABSI, which was clearly defined as one of the ten medical quality and safety improvement goals in 2021 by the CHN National Health Commission, is expected to get more resources and support [20]. In our opinion, this kind of practice that the government only sets goals but lacks unified steps or plans would have a certain effect in the short term, but this effect might be limited and unsustainable, because in the absence of supporting strategies (such as medical security, pay-for-performance appraisal, etc.), medical institutions were more willing to practice in a way suitable for the existing management mode, and evidence based guidelines would not be mandatory to be followed completely [21]. Taking the Centers for Medicare & Medicaid Services (CMS) in the United States as a guide [22], if the incidence of CLABSI could be used as an indicator to examine the reimbursement ratio of medical insurance or the pay-for-performance appraisal of hospitals, this goal might be easier to achieve in China.

We observed that PICCs were associated with a protective effect for CLABSI when compared to CVCs, regardless of adjusting for potential confounding factors. Yamaguchi et al. found similar results in a large sample of critically ill children [23]. Multiple reasons, including longer of length, lower of density in extremities bacteria, ease of insertion, and fewer number of lumen, accounted for the protective role of PICCs in CLABSI prevention [11, 23, 24]. Our study observed that PICCs have been used for a large proportion (around 80%). With the increasing use of PICC, it was easier to achieve the goal of reducing the incidence of CLABSI. Of note, in the first 4 calendar days after PICC insertion, there was a transient risk of PICC-associated BSI after puncture. As the trend reversed from days 5 through 8 after PICC insertion, the risk of PICC-associated BSI increased day by day from the 9th calendar day. Because of the trend reversal, the risk of PICC-associated BSI presents the characteristics of fluctuation curve. Sengupta et al. also found that the reversal trend occurred in neonates from days 19 through 35 after PICC insertion [25]. Although the specific reason for this curve feature was not clear, its existence might be one of the reasons why PICCs were associated with lower risk of CLABSI when compared to CVCs.

We observed a higher risk of CLABSI with CVCs than with PICCs after adjusting for several potential confounding factors. It was reasonable to focus more on CVCs in CLABSI prevention. The present study has shown that CLABSIs rates are differing among the three commonly used CVC insertion sites. Consistent with many previous studies [26, 27], CVC placement in the FEM site was associated with a substantially higher risk of CLABSI compared to the SC insertion and the IJ insertion. Notably, although avoidance of the FEM insertion was recognized to be very effective in reducing CLABSI rates and recommended by many guidelines, the FEM route was often chosen due to the ease and perceived lower insertion risk of this site [28]. Furthermore, before and after the 8th day of CVC intubation, the trend of CLABSI risk was different. Given the rapid increase in CLABSI risk during the first 8 calendar days after CVC insertion, we believe that this period should be considered an important period for infection control, and that substantial benefits could be obtained if the guidelines were well followed during this period. In contrast, the relative stable risk of CLABSI after the 8th day of CVC insertion meant that the benefits of CLABSI prevention might be lower than expected. Some clinical implications for this result could be highlighted. First, the first 8 calendar days after CVC insertion, the acute stage of CLABSI, were an important period of infection control, and it was worth doing our best to comply with standardized practices for the management of central lines. Most notably, compared with the study from Cobb and coworkers 30 years ago [29], the incidence rate of CVC-associated CLABSI was decreasing, but the acute phase might be prolonged. Second, routine replacement of CVCs with frequency of once every 8 days or longer interval did not prevent infection. In previous two trials, a strategy of changing the catheter every 7 days was used to assess the effect of scheduled catheter replacement, and there was no significant difference in their results [30, 31]. We suggested that this interval should be increased to 8 days in future trials.

Although evidence-based IPC guidelines have provided exact solutions to reduce the risk of CLABSI, how to make these methods operate effectively and maximize their effects should be highlighted. During our study period, the MDT team handled the problems that might hinder the implementation of IPC from the perspective of macro management, such as resource allocation, authorization management, verification, quality control and information monitoring. Consistent with the study from Jingjing Han, many systemic obstacles in China were considered to hold back evidence-based guidelines implementation exist, and the MDT CLABSI infection control program was considered to be effective in reducing hospital-wide CLABSI [32].

We acknowledge several limitations in our study. Most importantly, the CLABSI monitoring information system was the main way of data collection in this study. Although it helped the staff to realize the daily prospective monitoring of CLABSI, due to the initial design defects, some risk factors of CLABSI that had been confirmed in previous studies had not been collected (such as ICU admission now or in the past). Furthermore, our study was retrospective and only involved the analysis of a hospital. Larger multi-center randomized studies should be conducted to validate our findings.

## Conclusion

Our study found that PICCs have a protective role and the effect of fluctuation curve feature in CLABSI when compared to CVCs, and the first 8 calendar days after CVC insertion was the acute stage of CVC-associated CLABSI. CLABSI prevention was clearly defined as one of the ten medical quality and safety improvement goals in 2021 by the CHN National Health Commission. The popularization of PICC and intensive infection control in the acute stage of CVC-associated CLABSI would help to achieve this goal.

## Supplementary Information

**Additional file 1: Table S1. **Variable assignment table of baseline characteristics of our study population. **Table S2.** Bundled strategy for central line-associated bloodstream infections prevention and control (IPC).

## Data Availability

The datasets used and/or analysed during the current study are available from the corresponding author on reasonable request.
