# Supplementary material for: Peripherally inserted central catheters have a protective role and the effect of fluctuation curve feature in the risk of bloodstream infection compared with central venous catheters: a propensity-adjusted analysis
Source: BMC Infect Dis. 2022 Mar 26;22:289. doi: 10.1186/s12879-022-07265-x (PMC8961920; doi:10.1186/s12879-022-07265-x)
Supplement: Supplementary file 1 — Additional file 1: Table S1. Variable assignment table of baseline characteristics of our study population. Table S2. Bundled strategy for central line-associated bloodstream infections prevention and control (IPC). [file 12879_2022_7265_MOESM1_ESM.docx]

**Table S1.** Variable assignment table of baseline characteristics of our study population

| Variable name | Assignment |
| --- | --- |
| Male | 0 Female 1 Male |
| Year | 0 2017 1 2018 2 2019 3 2020 |
| Community Infections | 0 Unexposed 1 Exposed |
| Blood Transfusion | 0 Unexposed 1 Exposed |
| Urinary Catheterization | 0 Unexposed 1 Exposed |
| Hemodialysis | 0 Unexposed 1 Exposed |
| Mechanical Ventilation | 0 Unexposed 1 Exposed |
| Tracheotomy | 0 Unexposed 1 Exposed |
| Hypertension | 0 Unexposed 1 Exposed |
| Diabetes mellitus | 0 Unexposed 1 Exposed |
| COPD | 0 Unexposed 1 Exposed |
| Malignancy | 0 Unexposed 1 Exposed |
| Liver Failure | 0 Unexposed 1 Exposed |
| Renal Failure | 0 Unexposed 1 Exposed |
| Heart Failure | 0 Unexposed 1 Exposed |
| Respiratory Failure | 0 Unexposed 1 Exposed |
| Principal diagnosis (ICD-10 code) | Certain infectious diseases and parasites(A00-B99)  Tumor (C00-D48)  Blood and hematopoietic diseases and certain diseases involving immune mechanisms (D50-D89)  Endocrine, nutritional, and metabolic diseases (E00-E90)  Mental and behavioral disorders (F00-F99)  Nervous system diseases (G00-G99)  Eye and appendage diseases (H00-H59)  Ear and mastoid diseases (H60-H95)  Circulatory diseases (I00-I99)  Respiratory diseases (J00-J99)  Digestive diseases (K00-K93)  Skin and subcutaneous tissue diseases (L00-L99)  Musculoskeletal system and connective tissue diseases (M00-M99)  Genitourinary diseases (N00-N99)  Pregnancy, childbirth, and puerperium (O00-O99)  Congenital malformations, deformation, and chromosomal abnormalities (Q00-Q99)  Abnormal symptoms, signs, clinical and laboratory results, and cannot be classified in other categories (R00-R99)  Injury, poisoning and other external pathogenic factors (S00-T98)  External causes of illness and death (V01-V98) |

**Table S2.** Bundled strategy for central line-associated bloodstream infections prevention and control (IPC)

| **IPC measures** | **CVCs** | **PICCs** |
| --- | --- | --- |
| During and before intubation | 1. Prepare a complete set of items required for catheterization | 1. Prepare a complete set of items required for catheterization |
|  | 2. Daily 2% chlorhexidine skin cleansing | 2. Daily 2% chlorhexidine skin cleansing |
|  | 3. Hand hygiene | 3. Hand hygiene |
|  | 4. Maintain aseptic technique for the insertion of intravascular catheters | 4. Maintain aseptic technique for the insertion of intravascular catheters |
|  | 5. Sterile drape covering the patient's whole body | 5. Sterile drape covering the patient's whole body |
|  | 6. Alcohol defat and naturally dry | 6. Alcohol defat and naturally dry |
|  | 7. Chlorhexidine gluconate-ethanol skin disinfectant produced by 3M company was used during skin preparation. 2% tincture of iodine, 0.5% iodophor and 70% alcohol were used as alternatives in case of chlorhexidine gluconate allergy. | 7. Chlorhexidine gluconate-ethanol skin disinfectant produced by 3M company was used during skin preparation. 2% tincture of iodine, 0.5% iodophor and 70% alcohol were used as alternatives in case of chlorhexidine gluconate allergy. |
|  | 8. Personal protective equipment worn by the operator during puncture included disposable surgical masks, work caps, sterile surgical clothes and sterile gloves | 8. Personal protective equipment worn by the operator during puncture included disposable surgical masks, work caps, sterile surgical clothes and sterile gloves |
|  | 9. Use either sterile gauze or sterile, transparent, semipermeable dressing to cover the catheter site | 9. Use either sterile gauze or sterile, transparent, semipermeable dressing to cover the catheter site |
|  |  | 10.The outlet of the catheter was located at the upper middle of the elbow |
| After intubation | 1. Hand hygiene | 1. Hand hygiene |
|  | 2. Maintain aseptic technique for the care of intravascular catheters | 2. Maintain aseptic technique for the care of intravascular catheter |
|  | 3. The puncture point was clean and dry | 3. The puncture point was clean and dry |
|  | 4. There was no blood stain on the connecting port of the catheter | 4. There was no blood stain on the connecting port of the catheter |
|  | 5. Promptly remove any intravascular catheter that was no longer essential | 5. Promptly remove any intravascular catheter that was no longer essential |
|  | 6. Change the dressing in time | 6. Change the dressing in time |
|  | 7. Minimize contamination risk by scrubbing the access port with an iodophor and accessing the port only with sterile devices | 7. Minimize contamination risk by scrubbing the access port with an iodophor and accessing the port only with sterile devices |
